# Clinical outcome of pneumococcal meningitis during the emergence of pencillin-resistant *Streptococcus pneumoniae*: an observational study

**DOI:** 10.1186/1471-2334-11-323

**Published:** 2011-11-21

**Authors:** Edilane L Gouveia, Joice N Reis, Brendan Flannery, Soraia M Cordeiro, Josilene BT Lima, Ricardo M Pinheiro, Kátia Salgado, Ana Veronica Mascarenhas, M Gloria Carvalho, Bernard W Beall, Mitermayer G Reis, Albert I Ko

**Affiliations:** 1 Gonçalo Moniz Research Center, Oswaldo Cruz Foundation, Brazilian Ministry of Health, Salvador, Brazil; 2 School of Pharmacy, Federal University of Bahia, Salvador, Brazil; 3 Pan American Health Organization, Brasilia, Brazil; 4 Hospital Couto Maia, Secretary of Health for the State of Bahia, Salvador, Brazil; 5 Streptococcus Laboratory, Centers for Disease Control and Prevention, Atlanta, Georgia, USA; 6 Department of Epidemiology and Public Health, Yale School of Medicine, New Haven, USA

## Abstract

**Background:**

Prior to the availability of generic third-generation cephalosporins, penicillins were widely used for treatment of pneumococcal meningitis in developing countries despite concerns about rising levels of penicillin resistance among pneumococcal isolates. We examined the impact of penicillin resistance on outcomes of pneumococcal meningitis over a ten year period in an infectious diseases hospital in Brazil.

**Methods:**

Clinical presentation, antimicrobial therapy and outcomes were reviewed for 548 patients with culture-confirmed pneumococcal meningitis from December, 1995, to November, 2005. Pneumococcal isolates from meningitis patients were defined as penicillin-resistant if Minimum Inhibitory Concentrations for penicillin were greater than 0.06 μg/ml. Proportional hazards regression was used to identify risk factors for fatal outcomes.

**Results:**

During the ten-year period, ceftriaxone replaced ampicillin as first-line therapy for suspected bacterial meningitis. In hospital case-fatality for pneumococcal meningitis was 37%. Of 548 pneumococcal isolates from meningitis cases, 92 (17%) were resistant to penicillin. After controlling for age and severity of disease at admission, penicillin resistance was associated with higher case-fatality (Hazard Ratio [HR], 1.62; 95% Confidence Interval [CI], 1.08-2.43). Penicillin-resistance remained associated with higher case-fatality when initial therapy included ceftriaxone (HR, 1.68; 95% CI 1.02-2.76).

**Conclusions:**

Findings support the use of third generation cephalosporin antibiotics for treatment of suspected pneumococcal meningitis even at low prevalence of pneumococcal resistance to penicillins.

## Background

Among bacterial causes of meningitis, *Streptococcus pneumoniae *(the pneumococcus) is associated with the highest case-fatality and is the most likely to leave survivors with permanent sequelae. For the year 2000, the WHO's Global Burden of Disease project estimated 100,000 cases of pneumococcal meningitis occurred among children <5 worldwide, with higher than 50% case-fatality [1]. Over 95% of the pneumococcal deaths occurred in developing countries.

Prior to the identification of penicillin-resistant pneumococci, penicillin therapy was the standard treatment for pneumococcal meningitis. Recommendations of the American Academy of Pediatrics and Infectious Diseases Society of America for first-line treatment of pneumococcal meningitis were changed to a 3^rd ^generation cephalosporin [2-4], following reports of treatment failure and pharmacokinetic studies showing inadequate penicillin concentrations in the central nervous system to treat penicillin-resistant pneumococcal infections. However, the substantial difference in cost of third generation cephalosporins in the 1990s limited their use in developing countries. In 1997, the WHO recommended third generation cephalosporins as the first-line treatment for children ≥3 months of age with suspected bacterial meningitis in developed countries versus oily chloramphenicol and penicillin in developing countries [5], creating a double-standard [6-8]. The WHO recognized that where "significant" pneumococcal resistance to penicillin had been observed, cefotaxime or ceftriaxone were recommended, while leaving individual countries to determine what level of resistant would require a change in first-line therapy. At the time of these recommendations, few data from developing countries were available on the prevalence of penicillin-resistant pneumococci or its clinical relevance. Following availability of generic ceftriaxone after 1999, WHO recommended ceftriaxone alone as first-line antibiotic therapy for suspected bacterial meningitis in all countries [9]. However, despite availability of generic ceftriaxone, actual prices of this antibiotic may remain high and limit its use for treatment of pneumococcal meningitis in some developing countries [10].

To guide decisions about treatment of bacterial meningitis at an infectious diseases referral hospital in Salvador, Brazil, we established surveillance for penicillin-resistant pneumococcal infections in December 1995. The identification of penicillin resistance among 13% of pneumococcal isolates from meningitis cases during the first three years of surveillance led to a change in first-line antibiotic treatment to ceftriaxone [11]. Because data are limited on the clinical relevance of penicillin-resistance for outcomes of pneumococcal meningitis, we analyzed data from 10 years of surveillance for pneumococcal meningitis cases before and after introduction of empiric ceftriaxone therapy to investigate whether penicillin-resistance was associated with case-fatality and adverse outcomes of pneumococcal meningitis.

## Methods

### Patient population

Active hospital-based surveillance for bacterial meningitis was established in 1996 at Couto Maia Hospital, the state infectious diseases referral hospital in Salvador, Brazil (estimated population in 2000 of 2,443,107 inhabitants) [11]. The study protocol was approved by Institutional Review Boards of Couto Maia Hospital and the Oswaldo Cruz Foundation of the Brazilian Ministry of Health. Patients with suspected meningitis in the metropolitan region of Salvador were referred to Couto Maia hospital for initial diagnostic evaluation, including lumbar puncture and CSF analysis. After initial evaluation, patients were admitted or transferred (4%) to another hospital. The surveillance team reviewed the daily clinical laboratory record at the infectious disease hospital to identify all patients with an admission diagnosis of meningitis for whom *S. pneumoniae *were isolated from cerebrospinal fluid (CSF) or blood. Written informed consent was obtained for all patients to perform medical chart review and data collection. Antibiotic treatment decisions and use of systemic corticosteroids were made by hospital staff.

We limited the analysis of factors associated with case-fatality to pneumococcal meningitis cases with an isolate from blood or CSF and complete antimicrobial susceptibility testing. We included only the first episode of pneumococcal meningitis identified during the study period for 23 patients with recurrent episodes of pneumococcal meningitis (21 with two episodes, one with three and one with four). We also excluded three pneumococcal meningitis episodes with onset of symptoms during hospitalization. The analysis excludes 28 pneumococcal meningitis episodes (5 [18%] of which were caused by penicillin-resistant pneumococci) in patients who were transferred and for whom outcome was not known.

### Clinical data collection and definitions

For all identified patients with pneumococcal meningitis who were admitted to the surveillance hospital, a standardized entry form was used to extract demographic and clinical information from the medical records. Patients or members of their immediate family were interviewed to obtain information regarding duration of fever prior to hospitalization. Previous antibiotic use was defined as the use of any antibiotic in the previous month. Nosocomial infection was defined as hospitalization >3 days before the development of clinical symptoms of meningitis. Presence of underlying medical conditions were obtained by medical chart review or patient report and included AIDS, diabetes mellitus, asplenia, sickle cell disease, immunodepression and recurrent meningitis. Underlying conditions were assumed to be absent if not recorded in medical records. If not noted in clinical history or during physical examination at admission, patients were assumed not to have presented with seizures prior to admission or coma on admission.

Data were collected on antimicrobial treatment and corticosteroid use during hospitalization. According to Brazilian Ministry of Health guidelines [12], empiric treatment for suspected bacterial meningitis in patients 2 months to 5 years of age included ampicillin (200-400 mg/kg/24 hours) plus chloramphenicol (75-100 mg/kg/24 hours) or ceftriaxone alone (100 mg/kg/24 hours); ampicillin or ceftriaxone for patients older than 5 years; and ampicillin plus an aminoglycoside (gentamycin or amykacin; 15 mg/kg/24 hours), or cetriaxone plus ampicillin for infants less than 2 months of age. When ampicillin was not available, crystalline penicillin G (300,000 to 500,000 IU/kg every 4-6 h) was used in place of ampicillin. Antibiotic treatment was initiated as soon as possible. Initial ampicillin therapy was changed to ceftriaxone if the pneumococcal isolate demonstrated resistance to penicillin. For pneumococcal meningitis with reduced susceptibility to ceftriaxone, vancomycin (60 mg/kg/24 hours) was added. Discordant antibiotic therapy was defined as nonreceipt during the first twenty-four hours of hospitalization of any antibiotic to which the isolated *S. pneumoniae *was susceptible.

Patients were considered to have received corticosteroids only if administration began within the first two days of hospitalization. Sequelae were defined as presence at discharge of any neurological deficit, hearing loss or sensory deficit, which required evaluation by a neurologist or audiologist.

### Laboratory methods and definitions

Methods for the identification, serotyping and antimicrobial susceptibility testing of pneumococcal isolates were performed consistently throughout the study period as previously described [13]. Patient isolates were screened for reduced susceptibility to penicillin using a 1-ug oxacillin disk and penicillin Etest strips [11,14]; results were reported immediately to clinical staff. Minimal inhibitory concentrations (MIC) were defined according to the microdilution method as the lowest concentrations of antibiotics that inhibited pneumococcal growth *in vitro*. Microdilution trays were prepared according to the Clinical and Laboratory Standards Institute (CLSI; formerly the National Committee for Clinical Laboratory Standards) [15], including penicillin, chloramphenicol and cefotaxime for resistance to third-generation cephalosporins. For consistency with revised CLSI susceptibility breakpoints published in 2008 [15], we refer to pneumococcal meningitis isolates with a MIC > 0.06 μg/ml as penicillin-resistant (previously referred to as penicillin-nonsusceptible). We refer to isolates with MIC > 1 μg/ml as highly resistant to penicillins [15]. Resistance to chloramphenicol was defined as MIC > 4 μg/ml. Intermediate resistance to ceftriaxone was defined as MIC equal to 1 μg/ml.

### Statistical Analysis

A database of laboratory information was created and analyzed in Epi-Info, version 3.2 (Centers for Diseases Control and prevention, Atlanta, USA). Proportions of *S. pneumoniae *serotypes or nontypeable strains isolated from CSF or blood culture were compared with the χ^2 ^test with Yates' correction. We considered duration of symptoms prior to hospitalization, convulsions prior to admission, blood leukocyte count, CSF protein and glucose concentrations and presence of coma as potential markers of disease severity at admission. Generalized additive models (GAM) were used to determine threshold values to create dichotomous variables for blood leukocyte count, CSF protein and glucose. The association between covariates and the rate of death was analyzed in Cox proportional hazard regression models including age strata, gender and variables significantly associated with death in univariate analyses. Backward selection was used to identify a final model including the minimal set of statistically significant (p < 0.05) variables. We evaluated possible effect modification by age category by performing stratified multivariable analyses for patients <15 years versus those 15 years or older, and for patients initially treated with ceftriaxone versus those whose initial antibiotic therapy did not include ceftriaxone. All multivariate analyses were performed with R software (version 2.11.1; available at http://www.r-project.org).

## Results

We included 548 pneumococcal meningitis case patients in this analysis. Median age was 8 years (ranging from 0 to 79 years): 230 (42%) case patients were younger than 2 years of age (35% were younger than 1 year), and 6% were adults >50 years. Males comprised 64% (137/216) of cases in children <2 years, and 64% (354/548) overall. Underlying medical conditions were present in 141 (25.7%) patients and included: alcoholism (19), hypertension (16), repetition IVAS (12), chronic otitis media (12), diabetes (8), asthma (7), sickle cell anemia (6), and HIV infection (4). The presence of underlying disease was not associated with infection with penicillin-resistant pneumococci (Odds Ratio [OR] 0.6; 95% CI, 0.3-1.1]. Thirty-four patients had a history of prior hospitalization. Thirty-one patients had a history of meningitis before the episode included in the present study.

The median duration of symptoms before hospital admission was 2 days (mean: 2.9 range: 0 to 30 days). On presentation, 34% (181/533) of the patients had a history of generalized or focal seizures and 257 (48%) patients had altered mental status; of these, 32% (83/257) were in coma. The initial CSF parameters for these patients showed a mean leukocyte (WBC) count of 4,193/mm^3 ^(range 0 to 10,000). The mean protein and glucose concentrations were 357.3 and 26.2 mg/dl, respectively. Two hundred and thirty-five (42.8%) patients were admitted in ICU.

Of the 548 pneumococcal isolates from these patients, median MIC (MIC_50_) for penicillin was 0.03 μg/mL, 92 (17%) had MIC > 0.06 μg/mL and of these, only 3 were highly resistant (MIC >1.0 μg/mL); 63 (68%) of 92 penicillin-resistant pneumococci were isolated from children <2 years. The proportion of isolates resistant to penicillin ranged from 13 to 19% over the 10-year period, without evidence of an increasing trend (χ^2 ^for trend, p = 0.14) (Table 1). Three isolates with high-level penicillin resistance demonstrated intermediate resistance to cefotaxime. All isolates were susceptible to vancomycin, and 1% were resistant to chloramphenicol.

**Table 1:** Case fatality for confirmed pneumococcal meningitis by period (defined by empiric antibiotic therapy)

Characteristic	Time period ^a^						Total	
	Dec 1995--Nov 1998		Dec 1998--Nov 2002		Dec 2002--Nov 2005			
	N = 209		N = 220		N = 119			
	
	Cases	Deaths (% CFR)	Cases	Deaths (% CFR)	Cases	Deaths (% CFR)	Cases	Deaths (% CFR)
**Age category**								
<30 days	4	3 (75.0)	1	1 (100)	0	--	5	4 (80)
30-364 days	76	53 (69.7)	86	42 (48.8)	22	10 (45.4)	184	105 (57.1)
1-4 years	23	13 (56.5)	19	6 (31.6)	13	8 (61.5)	55	27 (49.1)
5-14 years	38	2 (5.3)	39	6 (15.4)	21	2 (9.5)	98	10 (10.2)
15-49 years	56	18 (32.1)	65	11 (16.9)	46	10 (21.7)	167	39 (23.3)
50 years or older	12	7 (58.3)	10	3 (30)	17	8 (47.1)	39	18 (46.1)
**Initial antibiotic therapy**								
Ampicillin or penicillin ^b^	124	46 (37.1)	40	6 (15.0)	5	0 (0)	169	52 (30.8)
Ceftriaxone	68	37 (54.4)	148	50 (33.7)	108	37 (34.3)	324	124 (38.3)
Ceftriaxone + Vancomycin	1	0 (0)	20	5 (25)	1	0 (0)	22	5 (22.7)
Other/Unknown	16	12 (75)	12	9 (75)	5	1 (20)	33	22 (66.7)
**Discordant therapy **^c^								
Yes	14	7 (50)	7	3 (42.9)	1	0 (0)	22	10 (45.5)
No	195	90 (46.2)	213	66 (30.9)	118	38 (32.2)	526	193 (36.7)
**Pneumococcal isolate resistant to penicillin**^d^	29	17 (58.6)	37	18 (48.6)	23	9 (39.1)	92	44 (47.8)

^a ^Study period was divided into three periods for the purposes of this analysis: empiric therapy containing a penicillin (December 1995-November 1998), transition period (December 1998-November 2002) and empiric therapy with ceftriaxone (December 2002-November 2005).

^b ^Includes regimens with ampicillin in combination with chloramphenicol or an aminoglycoside. A small proportion of patients received crystalline penicillin G in place of ampicillin.

^c ^Antibiotic therapy was defined as discordant when patients did not receive a β-lactam antibiotic for which the *S. pneumoniae *isolate was susceptible during the initial 24 hours of hospitalization.

^d ^Defined as penicillin MIC >0.06 μg/ml for a pneumococcal isolate from a patient with meningitis.

**Figure 1:**
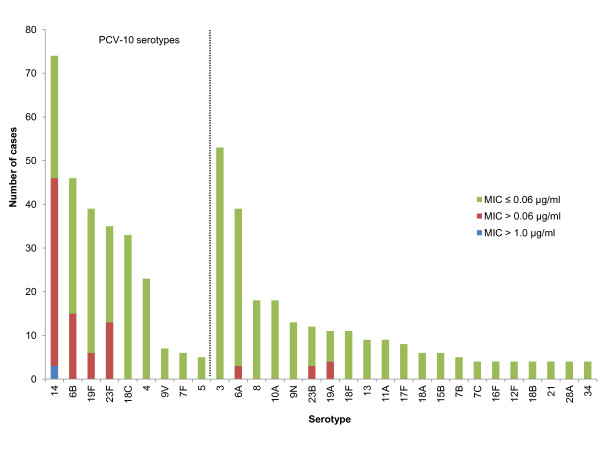
**Number of pneumococcal meningitis cases according to serotype and susceptibility of isolate to penicillin**. MIC, minimal inhibitory concentration determined by broth microdilution.

Serotypes included in the 10-valent and 13-valent pneumococcal conjugate vaccine accounted for 271 (49%) and 371 (68%) of 548 isolates from meningitis cases, respectively, and 80 (87%) versus 87 (95%) of 92 penicillin-resistant isolates (Figure 1). All three highly penicillin-resistant isolates were serotype 14. Among children <2 years, 152 (70%) and 120 (79%) of 216 isolates were serotypes included in the 10-valent or 13-valent vaccine, respectively.

**Figure 2:**
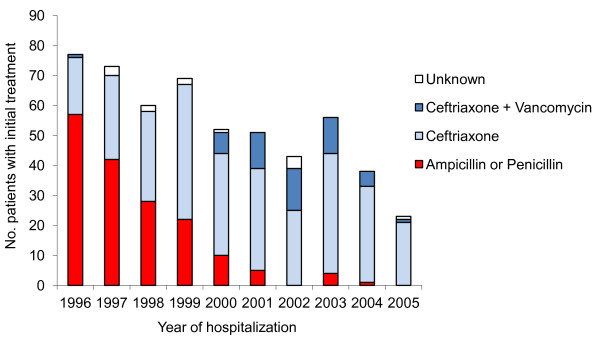
**Distribution of antibiotic used to treat patients during the study period**.

During the study period, we observed significantly declines in the proportion of pneumococcal meningitis cases treated initially with ampicillin (including 47 patients treated with ampicillin and chloramphenicol and few patients treatedwith penicillin G, χ^2 ^for trend = 155.7, p < 0.0001). Ceftriaxone alone became the first-line treatment for suspected bacterial meningitis, with vancomycin use rising and falling depending upon concerns about resistance to ceftriaxone (Figure 2). Systematic corticosteroids were administered on the day of admission to 412 (75%) of the patients. Most case patients received at least one antibiotic on the day of admission to which their isolate was susceptible (Table 1).

The overall case-fatality among hospitalized patients with pneumococcal meningitis was 37%: 57 patients (28% of deaths) died within 24 hours of admission, 55 (27%) died between 24 and 48 hours of admission, and a total of 91 patients (45%) died beyond 48 hours after hospital admission. Table 1 shows case-fatality proportions for meningitis cases stratified by age and time period. Case-fatality was highest during the first three years of the study, when empiric treatment for suspected bacterial meningitis included ampicillin (with or without chloramphenicol). However, 526 (96%) of 548 case patients received an initial antibiotic regimen to which their pneumococcal isolate was susceptible; only 22 (4%) initially received discordant therapy and of these, antibiotic therapy was changed within 48 hours of admission for 14 (64% of 22) patients. Children <5 years with penicillin-resistant infections tended to have longer duration of symptoms (median, 3 days) prior to hospitalization than those with penicillin-susceptible isolates (median, 2 days), although differences were not significant (p = 0.055). Among 7 patients with penicillin-resistant pneumococci who received discordant therapy, 4 died; 3 within 36 hours of admission and one patient 11 days after admission. One of the three patients who recovered had sequelae at discharge. Among the 10 patients who received discordant therapy and died, 6 died before 36 hours of admission; and 50% (5/10) were younger than 1 year old. Among 332 patients who received a neurological evaluation prior to discharge, 120 (36%) had a neurological deficit. The median duration of hospitalization was 15 days (range 3 to 90).

Infants and children younger than two years of age and patients 50 years and older had the highest proportions of case-fatality (Table 2). Female patients had a higher case-fatality (42%) than male patients (34%), both among patients younger than two years old (49 of 79 females [62%] versus 74 of 137 males [54%] died), and among patients two years or older (32 of 115 females [28%] versus 48 of 217 males [22%]). Patients who died had a significant lower blood leukocyte count, lower CSF leukocyte count and higher protein levels in CSF than survivors, but similar duration of symptoms prior to hospitalization (p = 0.5). Altered mental status upon admission and seizures prior to admission were associated with death. Underlying illness (presence of a chronic illness) was inversely associated with case-fatality in univariate analyses.

**Table 2:** Risk factors for death during hospitalization from pneumococcal meningitis in Salvador, Brazil

Characteristics	No. of cases	No. deaths (%)	Univariate Hazard Ratio (95% CI)	**Adjusted Hazard Ratio (95% CI) **^a^
**Age category**				
<1 years	189	109 (58)	2.58 (1.90-3.50)	2.61 (1.54-4.43)
1-4 years	55	27 (49)	2.20 (1.49-3.24)	3.14 (1.57-6.29)
5-14 years	98	10 (10)	0.46 (0.24-0.88)	0.67 (0.28-1.56)
15-50 years	170	38 (23)	1.0	1.0
>50 years	33	18 (55)	2.44 (1.61-3.71)	2.14 (1.11-4.10)
Female	193	81 (42)	1.21 (0.97-1.51)	
Underlying condition^b^	141	42 (30)	0.75 (0.57-1.00)	
**Clinical presentation**				
Seizures prior to hospitalization	181	83 (46)	1.40 (1.13-1.74)	
Coma on admission	83	47 (57)	1.69 (1.34-2.12)	2.55 (1.66-3.91)
**Laboratory values**				
Blood leukocyte count < 15,000 cells/μL	200	89 (44)	1.36 (1.09-1.69)	3.53 (2.18-5.71)
CSF protein >300 mg/dL^c^	308	125 (41)	1.36 (1.07-1.73)	1.88 (1.12-3.17)
Pneumococcal isolate resistant to penicillin^d^	93	45 (48)	1.39 (1.09-1.78)	1.62 (1.08-2.43)
Serotype 14	74	36 (49)	1.38 (1.06-1.80)	0.52 (0.29-0.91)
**Treatment**				
Discordant therapy ^e^	22	10 (45)	1.24 (0.77-1.98)	
Systemic corticosteroids ^f^	412/539	159 (39)	1.25 (0.94-1.68)	
ICU admission	235/548	130 (55)	2.37 (1.88-2.99)	

**NOTE**: CI, confidence interval; MIC, minimal inhibitory concentration; ICU, intensive care unit.

^a ^Hazard ratios and 95% confidence intervals from multivariable Cox proportional hazards model including age category, presence of coma, CSF protein >300 mg/dL, blood leukocyte count <15,000 cells/μL, penicillin MIC >0.06 μg/ml and serotype 14.

^b ^Includes HIV infection, diabetes mellitus, asplenia, sickle cell disease, congestive heart failure, other disorders associated with immunodepression and recurrent meningitis.

^c ^CSF protein measured at time of admission; data missing for 12 case patients.

^d ^Defined as penicillin MIC >0.06 μg/ml for a pneumococcal isolate from a patient with meningitis.

^e ^Antibiotic therapy was defined as discordant when patients did not receive any antibiotic to which the *S. pneumoniae *isolate was susceptible during the initial 24 hours of hospitalization.

^f ^Defined as initiation of corticosteroid therapy within 48 hours of admission; information missing for 9 case patients.

After adjusting for age group, penicillin-resistant pneumococcal meningitis cases had higher case-fatality rates than cases caused by penicillin-susceptible organisms. There were no significant differences in mean leukocyte count, protein or glucose concentrations in CSF at admission comparing patients with penicillin-resistant versus and penicillin-susceptible isolates. In multivariate analyses, risk factors for case-fatality were extremes of age, coma on admission, CSF protein >300 mg/dl, blood leukocyte count <15,000 cells/mm^3 ^and penicillin resistance (MIC>0.06 μg/mL) (Table 2). For each log_2 _increase in MIC, we observed a 20% increase in case-fatality (adjusted HR 1.20, 95% CI 1.06 to 1.36, p < 0.005). Compared to patients with pneumococcal isolates with MIC ≤ 0.03 μg/mL, those whose isolates had MICs of 0.06 or 0.12 μg/mL had nonsignificantly higher case-fatality (adjusted HR 1.40, 95% CI 0.89 to 2.19) and those whose isolates had MICs ≥ 0.25 μg/mL had significantly higher mortality (adjusted HR 2.65, 95% CI 1.38 to 5.05). Infection with pneumococcal serotype 14, the most frequent serotype among penicillin-resistant isolates, was associated with lower case-fatality after adjusting for age and penicillin-resistance (Table 2). In separate multivariable models for patients <15 years versus patients ≥ 15 years of age, mortality was significantly higher among patients < 15 years with penicillin-resistant isolates (adjusted HR 1.22, 95% CI 1.04 to 1.43), while the association did not reach statistical significance among older patients (adjusted HR 1.22, 95% CI 0.96 to 1.56). Corticosteroid use and discordant antibiotic therapy were not significant risk factors for death when included in the model. Excluding from the model 55 patients who died within 24 hours of hospitalization or 13 patients for whom initial antibiotic therapy was unknown did not alter the findings significantly. Penicillin-resistance remained associated with higher case-fatality when analysis was restricted to patients whose initial therapy included ceftriaxone (Table 3).

**Table 3:** Adjusted hazard ratios for death during hospitalization from final regression model stratified according to whether or not patient received initial ceftriaxone therapy.

Characteristics	Initial ceftriaxone therapy (N = 247)	Initial antibiotic other than ceftriaxone (N = 164)
	
	**Adjusted Hazard Ratio (95% CI) **^a^	
**Age category**		
<1 years	3.44 (1.58-7.48)	2.55 (1.21-5.39) ^b^
1-4 years	4.16 (1.67-10.38)	
5-14 years	2.61 (0.86-7.94)	1.0^b^
15-50 years	1.0	
>50 years	1.72 (0.63-4.68)	2.61 (1.07-6.39)
**Clinical presentation**		
Coma on admission	2.42 (1.36-4.30)	2.82 (1.43-5.56)
**Laboratory values**		
Blood leukocyte count < 15,000 cells/μL	4.38 (2.33-8.25)	2.68 (1.26-5.69)
CSF protein >300 mg/dL^c^	2.72 (1.44-5.14)	1.19 (0.45-3.15)
Pneumococcal isolate resistant to penicillin^d^	1.68 (1.02-2.76)	1.37 (0.65-2.89)
Serotype 14	0.48 (0.25-0.90)	0.52 (0.14-1.93)

**NOTE**: CI, confidence interval; MIC, minimal inhibitory concentration.

^a ^Hazard ratios and 95% confidence intervals from multivariable Cox proportional hazards model including age category, presence of coma, CSF protein >300 mg/dL, blood leukocyte count <15,000 cells/μL, penicillin MIC >0.06 μg/ml and serotype 14.

^b ^For patients who received initial antibiotic therapy other than ceftriaxone, age categories for <1 and 1-4 years, and for 5-14 and 15-50 years were combined.

^c ^CSF protein measured at time of admission; data missing for 12 case patients.

^d ^Defined as penicillin MIC >0.06 μg/ml for a pneumococcal isolate from a patient with meningitis.

## Discussion

In this large series of pneumococcal meningitis cases prospectively identified over a ten year period of active surveillance at a public infectious disease referral hospital in Brazil, we found an association between case-fatality and penicillin resistance among pneumococcal meningitis cases. This association is unlikely to be due to decreased sensitivity to penicillin alone, because few patients with penicillin resistant isolates received initial penicillin treatment. The small number of patients who received discordant therapy, or antibiotics to which their isolate was resistant, resulted in low statistical power to detect an independent effect of discordant therapy on outcome. In addition, initial penicillin therapy was changed to ceftriaxone once results of antibiotic susceptibility testing showed that the isolate had reduced sensitivity to penicillin. During the study period, ceftriaxone replaced penicillin as first-line therapy for suspected bacterial meningitis, yet we did not find that initial treatment with ceftriaxone reduced case-fatality. Young children, with the highest case-fatality, were more likely to have penicillin-resistant pneumococcal isolates. Following this initial finding, the hospital purchased limited amounts of cefriaxone for empiric treatment of young children with suspected bacterial meningitis. Because the majority of deaths occurred soon after admission, initial ceftriaxone therapy may not significantly have improved survival; patients with penicillin resistant pneumococcal infections may have presented with more severe disease. Alternatively, although we controlled for age in the analysis, young age may still have confounded the association between case-fatality and infection with penicillin-resistant pneumococci.

Prior to the availability of low cost third-generation cephalosporins, developing countries faced a dilemma in dealing with the problem of rising penicillin resistance among *S. pneumoniae*: implement empiric therapy with cephalosporins at substantial increased cost to the health system, or maintain empiric therapy with penicillin (and chloramphenicol) [6]. Treatment of resistant pneumococcal infections with chloramphenicol and penicillin risked treatment failure and adverse outcomes, even at high concentrations of penicillin [16-18]. Pharmacokinetic studies indicated poor penetration of penicillin into cerebrospinal fluid, which resulted in concentrations below those required for sterilization of CSF for pneumococcal isolates with MIC > 0.06 μg/mL [19]. In addition, failure of chloramphenicol treatment has been described in patients infected with penicillin-resistant pneumococci [17]. In settings in which antimicrobial susceptibility testing was available, patients with penicillin-resistant pneumococcal infections could receive appropriate treatment after results of susceptibility testing were known. In practice, this strategy leads to delay in administration of appropriate treatment, which is associated with delayed sterilization of CSF and worse outcomes [20]. Delays in diagnosis have also been associated with poor outcomes in bacterial meningitis [21] and likely contributed to the high case-fatality in our setting. An alternative strategy used successfully in Papua New Guinea initiated empiric therapy with ceftriaxone and switched to a less expensive antibiotic (chloramphenicol) once isolates were identified as fully susceptible [22]. In our setting, switching from ceftriaxone to penicillin for penicillin-sensitive isolates was uncommon due to a preference for administration of ceftriaxone at 12-hour intervals versus 4- or 6-hour intervals for penicillin G or ampicillin, respectively.

Our findings underscore the importance of antimicrobial susceptibility testing, both to guide treatment as well as to provide local data on prevalence of antimicrobial resistance. Following identification in 1996 of a cluster of penicillin-resistant pneumococcal meningitis cases with high case-fatality [11], the infectious diseases hospital increased its purchase of ceftriaxone, at the time ten times the cost of penicillin therapy. Initially, ceftriaxone was prioritized for treatment of children due to higher proportions of penicillin resistant pneumococcal meningitis among children. One of the objectives of monitoring outcomes of pneumococcal meningitis cases at the study hospital was to justify the increased expense of empiric ceftriaxone therapy. However, despite evidence of increasing resistance to penicillin from several settings in Brazil [23,24], the Brazilian Ministry of Health did not change recommended first-line therapy for suspected bacterial meningitis or pneumococcal meningitis to ceftriaxone due to concerns about costs and risk of stock-outs of the more expensive antibiotic. The prevalence of penicillin resistant infections was considered low enough not to warrant revision of national recommendations, acknowledging the need for rapid identification of resistant infections and local decisions regarding choice of empiric therapy. One result of the WHO recommendations was that Ministries of Health in developing countries were left to make their own decisions regarding levels of penicillin resistance considered acceptable for use of penicillin as first-line therapy for pneumococcal meningitis [5]. However, few developing countries had representative data on antimicrobial susceptibility patterns among invasive pneumococcal isolates.

The 2008 revision of Clinical and Laboratory Standards Institute susceptibility breakpoints for *Streptococcus pneumoniae *brought laboratory definitions in line with treatment recommendations [15]. Prior to the 2008 revision, invasive pneumococcal isolates with penicillin MIC > 0.06 μg/mL were classified as penicillin-nonsusceptible: penicillin MIC from 0.1 to 1.0 μg/mL was classified as intermediate and MIC > 1.0 μg/ml as resistant [25]. Observational studies suggested that high dose penicillin therapy was effective for treatment of invasive pneumococcal infections outside the central nervous system [26]. Together with the limited clinical data on outcomes of penicillin-resistant pneumococcal meningitis treated with penicillin, this classification created uncertainty regarding the treatment of pneumococcal meningitis with intermediate resistance to penicillin. The low prevalence of pneumococcal isolates with MIC > 1.0 μg/mL in studies throughout Brazil [27] was considered by the Brazilian Ministry of Health not to justify replacement of penicillin as first-line therapy for suspected bacterial meningitis.

Additional data are needed on potential benefits and risks of adjunct dexamethasone therapy for pneumococcal meningitis in developing countries, especially in settings where antibiotic resistance is prevalent. In a 2010 Cochrane review, adjunctive treatment with dexamethasone was associated with lower case-fatality in pneumococcal meningitis and lower incidence of severe hearing loss in children with meningitis due to *Haemophilus influenzae*, but no benefit of dexamethasone treatment was found for bacterial meninigitis patients in low-income countries [28]. One randomized, controlled trial conducted in six Latin American countries showed that treatment with oral glycerol reduced neurological sequellae in children with meningitis, suggesting alternatives to dexamethasone treatment [29]. Brazilian Ministry of Health guidelines for treatment of bacterial meningitis provide no recommendation regarding use of corticosteroids [12]. In our analysis, we observed no confounding effect of adjunct corticosteroid therapy on the association between case-fatality and penicillin resistance. Although the use of steroids was frequent among patients with pneumococcal meningitis, dexamethaxone was not used systematically and was indicated according to physician preference. Therefore, the lack of association between initial dexamethasone therapy and outcome of pneumococcal meningitis in this series should be interpreted with caution, as with other observational studies [30]. Concern has been expressed that treatment with steroids would reduce the vancomycin concentrations in cerebrospinal fluid in adults and therapeutic failures have been described [30]. In our hospital, the pneumococcal meningitis cases treated with ceftriaxone and vancomycin were mainly pediatric; most also received dexamethaxone.

The availability of low cost ceftriaxone therapy in developing countries makes the findings of this study less relevant for decisions regarding choice of empiric therapy for suspected bacterial meningitis or pneumococcal meningitis. However, surveys of essential medicines in developing countries show wide variability in the actual price of ceftriaxone and its availability in public hospitals, suggesting that penicillins may still be used for first-line treatment in some countries [10]. Pneumococcal resistance to cephalosporins needs to be monitored [31,32], although cefotaxime nonsusceptibility has not been associated with increased mortality of pneumococcal meningitis treated with cefotaxime [33-35].

The high mortality and burden of pneumococcal meningitis among Brazilian children were among the principal reasons for the Brazilian Ministry of Health's decision to introduce pneumococcal conjugate vaccine in the routine infant immunization schedule in 2010. Brazil was one of the first countries to introduce a novel pneumococcal conjugate vaccine including 10 serotypes into its national immunization program. Based on data from this surveillance as well as laboratory-based surveillance in Brazil [36], the 10-valent vaccine formulation includes the most prevalent serotypes in pneumococcal meningitis cases in young children, including those serotypes accounting for the majority of penicillin-resistant isolates. Because the 10-valent vaccine was licensed based on immunogenicity data, its effectiveness against invasive pneumococcal disease due to vaccine serotypes, as well as its effect on potentially cross-reactive serotypes (such as 6A and 19A) needs to be determined. Antibiotic use may exert selective pressure leading to expansion of resistant clones with non-vaccine type capsular polysaccharides. Continued surveillance following vaccine introduction is warranted to monitor antibiotic resistance among invasive pneumococcal infections to guide treatment decisions.

## Conclusions

In this observational study of pneumococcal meningitis outcomes, patients infected with pneumococci with reduced susceptibility to penicillin had higher case-fatality than patients with penicillin-sensitive isolates, even when initial antibiotic therapy included ceftriaxone. Appropriate antibiotic therapy may not have been initiated early enough in the course of infection to prevent death. Although ceftriaxone has become more available in developing countries since the time of this study, these findings are relevant where penicillin antibiotics are still used due to lack of information about penicillin resistance or limited availability of ceftriaxone. These findings support the use of third generation cephalosporin antibiotics for treatment of suspected pneumococcal meningitis even at low prevalence of pneumococcal resistance to penicillins.

## Competing interests

The authors declare that they have no competing interests.

## Authors' contributions

ELG, JNR, MGR and AIK designed the study. ELG, RMP, KS, AVM and AIK collected clinical and laboratory data. JNR, SMC, KS and JBTL performed pneumococcal characterization and antimicrobial susceptibility testing. MGC and BB participated in pneumococcal serotyping. ELG, JNR, BF and AIK analyzed and interpreted the data. ELG, JNR and AIK drafted the manuscript. All authors read and approved the final manuscript.

## Pre-publication history

The pre-publication history for this paper can be accessed here:

http://www.biomedcentral.com/1471-2334/11/323/prepub
